# A Plasma Circular RNA Profile Differentiates Subjects with Alzheimer’s Disease and Mild Cognitive Impairment from Healthy Controls

**DOI:** 10.3390/ijms232113232

**Published:** 2022-10-31

**Authors:** Paola Piscopo, Valeria Manzini, Roberto Rivabene, Alessio Crestini, Loredana Le Pera, Elisabetta Pizzi, Caterina Veroni, Giuseppina Talarico, Martina Peconi, Anna Elisa Castellano, Carmelo D’Alessio, Giuseppe Bruno, Massimo Corbo, Nicola Vanacore, Eleonora Lacorte

**Affiliations:** 1 Department of Neuroscience, Istituto Superiore di Sanità, 00161 Rome, RM, Italy; 2 EBRI Rita Levi-Montalcini Foundation, 00161 Rome, RM, Italy; 3 Servizio Grandi Strumentazioni e Core Facilities, Istituto Superiore di Sanità, 00161 Rome, RM, Italy; 4 Department of Human Neuroscience, University of Rome “Sapienza”, 00185 Rome, RM, Italy; 5 Department of Neurology, IRCCS Neuromed Institute, 86077 Pozzilli, IS, Italy; 6 Department of Neurorehabilitation Sciences, Casa Cura Policlinico, 20144 Milan, MI, Italy; 7 National Center for Disease Prevention ad Heath Promotion, Istituto Superiore di Sanità, 00162 Rome, RM, Italy

**Keywords:** circular RNA, circRNA, Alzheimer Disease, AD, mild cognitive impairment, MCI, biomarkers, microarray Real-Time PCR

## Abstract

The most frequently used biomarkers to support the diagnosis of Alzheimer’s Disease (AD) are Aβ42, total-Tau, and phospho-tau protein levels in CSF. Moreover, magnetic resonance imaging is used to assess hippocampal atrophy, 18F-FDG PET to identify abnormal brain metabolism, and PET imaging for amyloid deposition. These tests are rather complex and invasive and not easily applicable to clinical practice. Circulating non-coding RNAs, which are inherently stable and easy to manage, have been reported as promising biomarkers for central nervous system conditions. Recently, circular RNAs (circRNAs) as a novel class of ncRNAs have gained attention. We carried out a pilot study on five participants with AD and five healthy controls (HC) investigating circRNAs by Arraystar Human Circular RNA Microarray V2.0. Among them, 26 circRNAs were differentially expressed (FC ≥ 1.5, *p* < 0.05) in participants with AD compared to HC. From a top 10 of differentially expressed circRNAs, a validation study was carried out on four up-regulated (hsa_circRNA_050263, hsa_circRNA_403959, hsa_circRNA_003022, hsa_circRNA_100837) and two down-regulated (hsa_circRNA_102049, hsa_circRNA_102619) circRNAs in a larger population. Moreover, five subjects with mild cognitive impairment (MCI) were investigated. The analysis confirmed the upregulation of hsa_circRNA_050263, hsa_circRNA_403959, and hsa_circRNA_003022 both in subjects with AD and in MCI compared to HCs. We also investigated all microRNAs potentially interacting with the studied circRNAs. The GO enrichment analysis shows they are involved in the development of the nervous system, and in the cellular response to nerve growth factor stimuli, protein phosphorylation, apoptotic processes, and inflammation pathways, all of which are processes related to the pathology of AD.

## 1. Introduction

Alzheimer’s disease is currently diagnosed by clinical evaluation with the support of magnetic resonance imaging [1], 18F-FDG PET [2], and Amyloid PET, which evaluates neurodegeneration, brain metabolism, and Amyloid deposition [3,4]. In addition, cerebrospinal fluid (CSF) biomarkers analysis, such as Aβ42, total-tau, and phospho-tau [5] are also used. These tests are rather complex and, above all, invasive, so the associated procedures are not easily applicable to clinical practice. In this context, there is therefore a need to identify new non-invasive and easy-to-monitor biomarkers.

Numerous studies are currently focused on blood, plasma, and serum biomarkers, such as microRNAs (miRNAs). They are linear small protein-associated non-coding RNAs, which are easy and cheap to detect thanks specifically RNA purification techniques. Nevertheless, the main methods of RNA detection were built on the separation of only linear RNA molecules, often based on polyadenylated (poly-A) tails recognition, hiding for a long time all those RNA species without poly-A ends. Among these, circular RNAs appeared to be particularly interesting, abundant, and widespread.

Circular RNAs (circRNAs) are a class of non-coding RNAs, about 100–1000 nucleotides long, characterized by a single-strand covalently closed circular structure, without cap and poly-A tail. Some RNA polymerase II-derived transcripts, independent or linear RNA-linked, might undergo back-splicing events that produce endogenous non-coding circRNAs [6]. Their main function is to regulate miRNA expression levels and activities. In particular, they act as sponges that capture miRNAs preventing their post-transcriptional repressive effects on messenger RNA (mRNAs) targets, consequently regulating the expression of the same mRNAs [6,7]. Recent studies described circRNAs as molecules implicated in the AD pathological process, suggesting a potential role in the pathogenesis of this disease [8].

CircRNAs are very abundant in plasma, sometimes even 10 times more than mRNA, because their circular structure can escape the RNases-mediated degradation. In fact, the RNases are able to recognize linear RNA ends that are not a characteristic of circRNAs [9]. The process by which they are released into the extracellular space is still unknown, but their presence in the blood is easy to detect with quantitative real-time polymerase chain reaction (qRT-PCR), microarray, or deep sequencing. Moreover, they have great tissue and cellular stage specificities, reflecting their role in physiological or pathological development. A large portion of plasmatic circRNAs is derived from the central nervous system (CNS). Theoretically, available technologies could even allow identifying the specific cerebral area they are derived from [10]. In addition, their high conservation between species allows a relative comparison between the circRNAs from animal and human models [6]. On this basis, circRNAs have a high potential to become possible diagnostic biomarkers.

In this study, we focused on circRNAs in plasma samples of people with AD and healthy controls (HC) to investigate their possible utility as biomarkers. We profiled differentially expressed circRNAs using circRNA microarrays. The validation with qRT-PCR on a larger population confirmed our results. Our analysis was extended to a small cohort of MCI subjects as a promising preliminary study. We investigated all microRNAs potentially interacting with the selected circRNAs and performed Gene ontology (GO) and Kyoto Encyclopedia of Genes and Genomes (KEGG) pathway analyses.

## 2. Results

### 2.1. CircRNAs Profile by Microarray

We hypothesized that circRNAs could be differentially expressed among people with AD and healthy controls. To verify our hypothesis, we performed a pilot study on a small population (Table 1): plasma samples from five participants with AD (mean age 74.4 ± 2.3, two males and three females) and five healthy controls (mean age 73.8 ± 2.3, two males and three females) were analyzed by microarrays to identify all differentially expressed circRNAs. All samples were quality checked, and they passed the test. Thus, they were prepared according to manufacturer protocol and hybridized on Arraystar Human circRNA Array V2 (8×15K, Arraystar). The used microarrays were able to investigate the presence of 13,617 different circRNAs. CircRNAs were divided into five groups: 1. “Exonic”, from the exons of the linear transcripts; 2. “Intronic”, from introns of the linear transcripts; 3. “Antisense”, with gene loci overlapping with linear RNAs, but transcribed from opposite strands; 4. “Sense overlapping”, transcribed from the same gene loci as linear transcripts, but not classified as “exonic” or “intronic”; 4. “Intergenic”, located outside known gene loci.

Out of the 13,617 interrogated types of probes, 10,147 circRNAs (74.52%) were detected by the microarray signals as significantly above the background in the samples of at least one of the groups. The differentially expressed circRNAs (*p* < 0.05) were 26: 3 downregulated and 23 upregulated, as shown in Table 2. Among them, the ones with higher fold change (FC ≥ 2.0) and lower *p*-values were selected for further analyses. 

The circRNA expression values were normalized and graphed (Figure S1A) in a scatter plot, while the volcano plot shows a significant difference between the two different groups (Figure S1B). Additionally, a box plot view was used to compare the distribution of the dataset from the samples after normalization (Figure S1C).

Moreover, cluster analysis arranged samples into groups, allowing us to hypothesize about the relationship among samples. The hierarchical cluster was performed on the differentially expressed circRNAs (Figure S1D). This clustering plot shows that the circRNA expression pattern can classify individuals according to their status, except for one healthy control, who is grouped with Alzheimer’s Disease.

### 2.2. Validation of Candidate circRNAs by qRT-PCR

For the validation, we extended our study to a larger population of 24 HC (mean age 70.0 ± 6.6, 10 males and 14 females) and 21 participants with AD (mean age 74.4 ± 6.1, mean age at onset 70.6 ± 6.8, 9 males and 12 females). We also investigated five subjects with MCI (mean age 70.0 ± 7.3, three males and two females) following the same protocol, as reported in Table 1. The CSF biomarkers for AD and MCI subjects did not available, thus we performed the innovative Simoa assay to assess Total Tau, phosphorylated in 181 Tau, Amyloid beta 40, and Amyloid beta 42 peptides in plasma samples. We evaluated a possible correlation between these biomarkers and circRNAs, but we did not find any significant result (data not shown).

**Figure 1:**
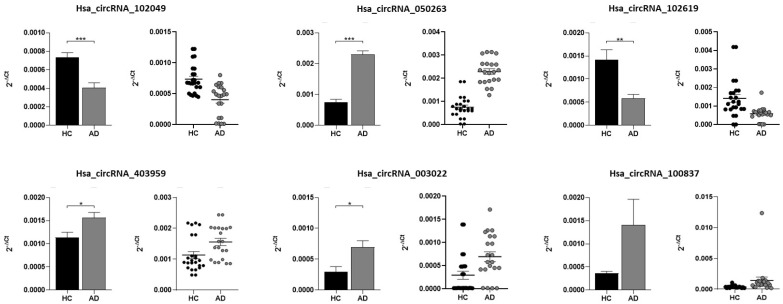
Graphic representation and Scatter plots of circRNAs levels between AD patients and HCs. * *p* < 0.05, ** *p* < 0.01, *** *p* < 0.001.

We selected 10 dysregulated circRNAs, out of which eight were upregulated (hsa_circRNA_100760, hsa_circRNA_001131, hsa_circRNA_405788, hsa_circRNA_407191, hsa_circRNA_050263, hsa_circRNA_403959, hsa_circRNA_003022, hsa_circRNA_100837), and two were downregulated (hsa_circRNA_102049, hsa_circRNA_102619), based on their *p* values, fold changes and signal intensities. The expression levels of hsa_circRNA_100760, hsa_circRNA_001131, hsa_circRNA_405788, and hsa_circRNA_407191 were not detectable in plasma samples through qRT-PCR. However, we obtained data for the remaining six circRNAs (Table 1). Five of them were significantly differentially expressed (Figure 1). Consistent with microarray data, in patients with AD the expression of hsa_circRNA_050263, hsa_circRNA_403959, hsa_circRNA_003022 was significantly higher, while the expression of hsa_circRNA_102049, hsa_circRNA_102619 was significantly lower compared to HCs. When we adjusted for sex and age, the statistical significance remained, except for hsa_circRNA_003022 (*p* = 0.094). No significant differences in the expression of hsa_circRNA_100837 were observed, even if their *p* reached a value near to significance when it was adjusted for sex and age (*p* = 0.072)

**Figure 2:**
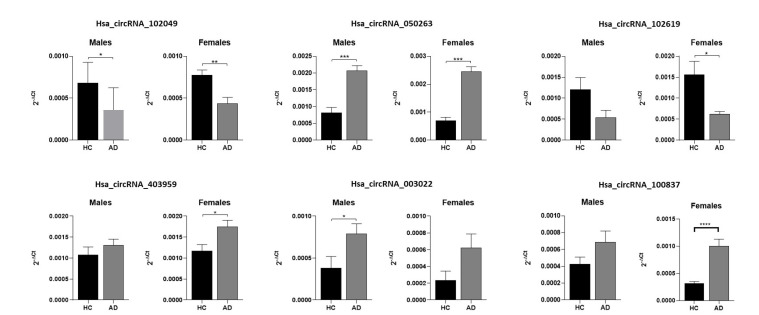
Graphic representation of circRNA expression levels in males and females in subjects with HC and AD. * *p* < 0.05, ** *p* < 0.01, *** *p* < 0.001, **** *p* < 0.0001.

When stratifying all subjects by gender, we noted significant differences in the levels of circRNAs between AD and HCs both in males and in females in circRNA_102049 and circRNA_050263. On the contrary, circRNA_102619 and circRNA_403959 were differentially expressed only in females, while circRNA_03022 was upregulated only in males (Figure 2). Interestingly, statistically significant overexpression of circRNA_100837 was observed in female participants with AD compared to HCs, with non-significant data in the whole population.

**Figure 3:**
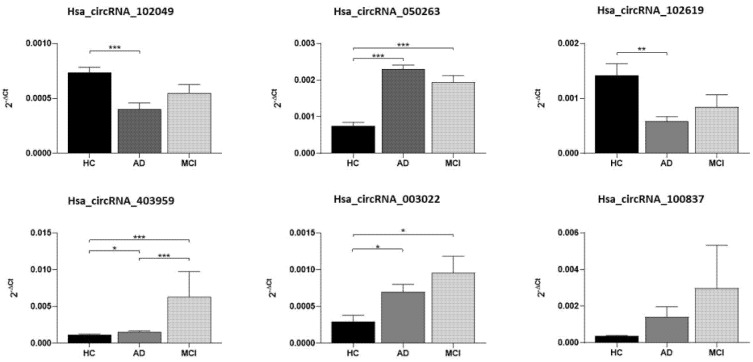
Graphic representation of circRNA expression levels in HC, AD, and MCI subjects. * *p* < 0.05, ** *p* < 0.01, *** *p* < 0.001.

In the validation study, we added a small population of subjects with MCI, and we found upregulated levels similar to AD for hsa_circRNA_050263, hsa_circRNA_403959, hsa_circRNA_003022 relative to HCs (Figure 3). When we adjusted for sex and age, the statistical significance remained, and for circRNA_100837 the *p* reached a value near significance (*p* = 0.055). Table 3 summarizes the circRNAs results of the validation study.

### 2.3. ROC Showed Good Diagnostic Accuracy for All Six miRNAs Analyzed

**Figure 4:**
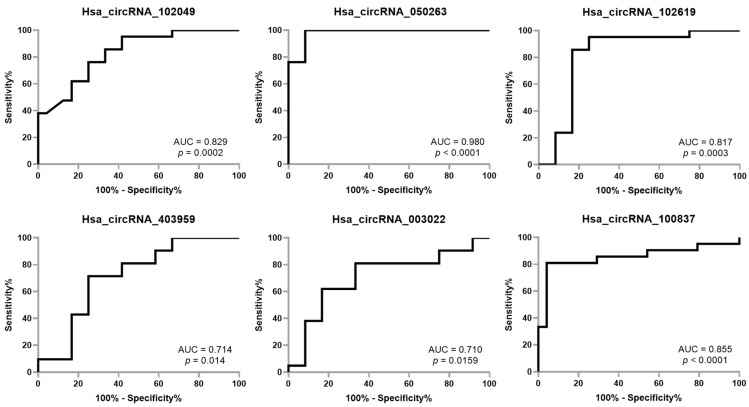
Receiver operating characteristic (ROC) curve of differentially expressed circRNAs in AD vs HC. AUC: Area Under the Curve.

To determine the diagnostic accuracy of the six circRNAs as possible biomarkers, we performed ROC curve analyses. We observed that hsa_circRNA_050263 could distinguish healthy controls from AD patients with an excellent diagnostic accuracy (AUC: 0.98, *p* < 0.0001), hsa_circRNA_102619 (AUC: 0.817, *p* = 0.0003), hsa_circRNA_102049 (AUC: 0.829, *p* = 0.0002) and hsa_circRNA_10083749 (AUC: 0.855, *p* < 0.0001) with a good accuracy, and hsa_circRNA_003022 (AUC: 0.710, *p* = 0.0159) and hsa_circRNA_403959 (AUC: 0.714, *p* = 0.014) with a fair accuracy (Figure 4).

### 2.4. CircRNA/miRNA Network

**Figure 5:**
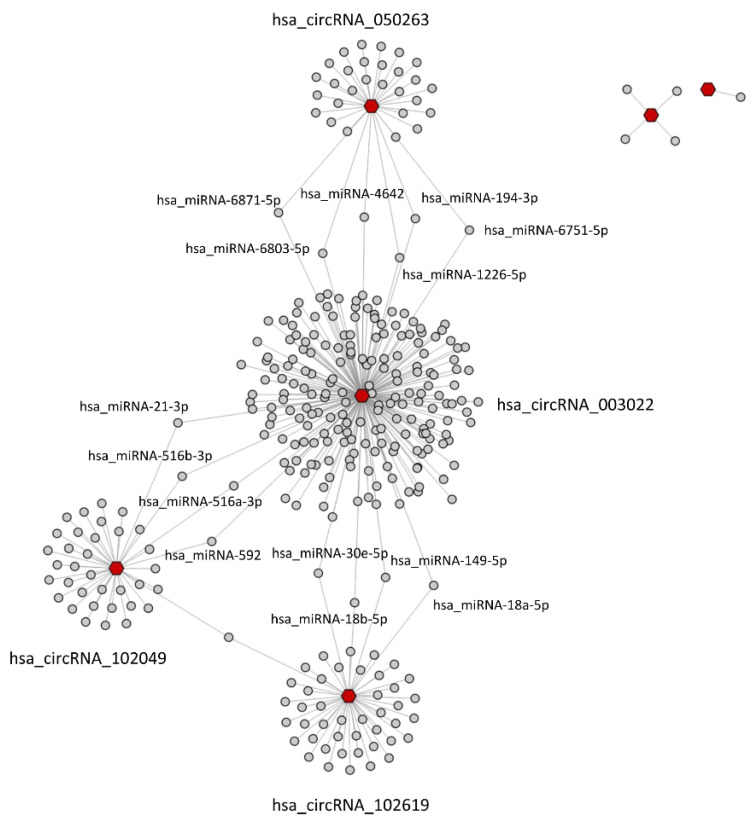
circRNA-miRNA network was reconstructed by Cytoscape 3.9.0. Red dots represent hasa_circRNAs, whilst hsa_miRNAs are shown as grey dots. Hsa_miRNAs functioning as bridges between hsa_circ_RNAs clusters are labeled.

The six differentially expressed circRNAs under investigation were further analyzed to predict their potential miRNA targets using the TargetScan [11] and miRanda [12] software. A total of 331 miRNAs were identified as potential target of the six circRNAs (Table S1). Only 15 miRNAs were found to be the targets of more than one circRNA (hsa-miR-18b-5p, hsa-miR-149-5p, hsa-miR-30e-5p, hsa-miR-18a-5p, hsa-miR-197-5p, hsa-miR-6871-5p, hsa-miR-6751-5p, hsa-miR-194-3p, hsa-miR-6803-5p, hsa-miR-622, hsa-miR-21-3p, hsa-miR-592, hsa-miR-516a-3p, hsa-miR-516b-3p, hsa-miR-4441). Each circRNA essentially appears to have specific miRNAs, not shared with other circRNAs, as evidenced by the representation of the circRNA-miRNA network in Figure 5. In particular, hsa_circRNA_403959 and hsa_circRNA_100837 were disconnected nodes of the network, and both of them collected a few potential target miRNAs. Therefore, the following analyses focused on characterizing the potential functional roles of the other four circRNAs, which appear to have a greater weight within the circRNA-miRNA-mRNA regulatory network. 

**Figure 6:**
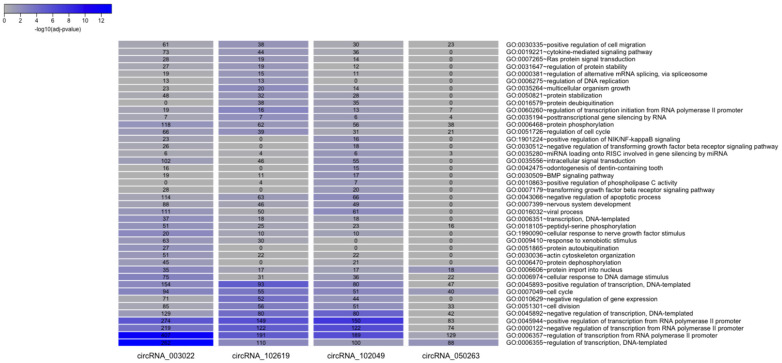
Comparison of Biological Processes GO terms over-represented in the groups of target proteins of each differentially expressed circRNA. All terms statistically enriched in at least one of the four groups (circRNA_003022, circRNA_102619, circRNA_102049, circRNA_050263) are reported in the heatmaps, with adjusted *p*-values plotted in blue-grey scale color, where blue indicates higher significant results.

**Figure 7:**
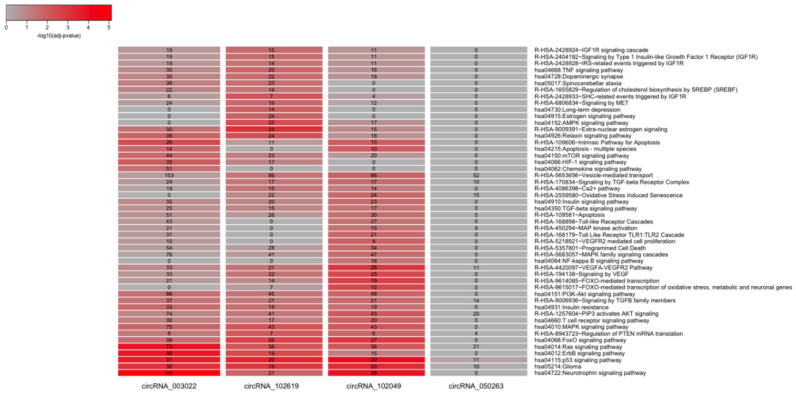

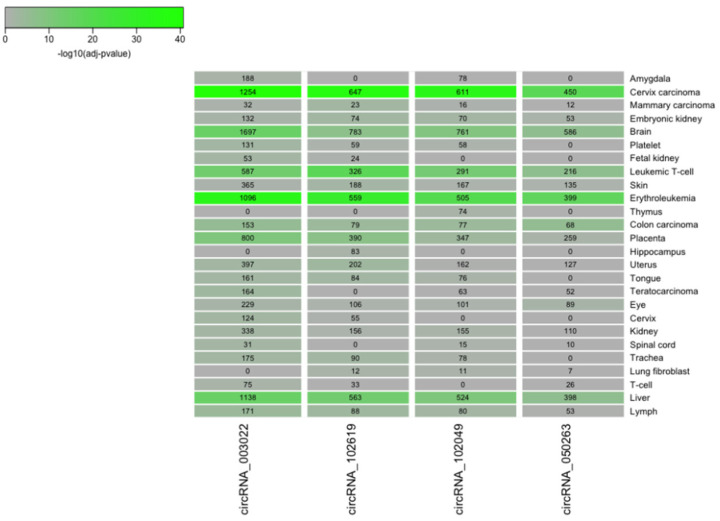
Comparison of KEGG and Reactome pathways (on the **top**) and tissues (on the **bottom**) significantly over-represented in the annotations of groups of target proteins of each differentially expressed circRNA. All terms statistically enriched in at least one of the four groups (circRNA_003022, circRNA_102619, circRNA_102049, circRNA_050263) are reported in the heatmaps, with adjusted *p*-values plotted in red- and green-grey color scales, respectively.

**Figure 8:**
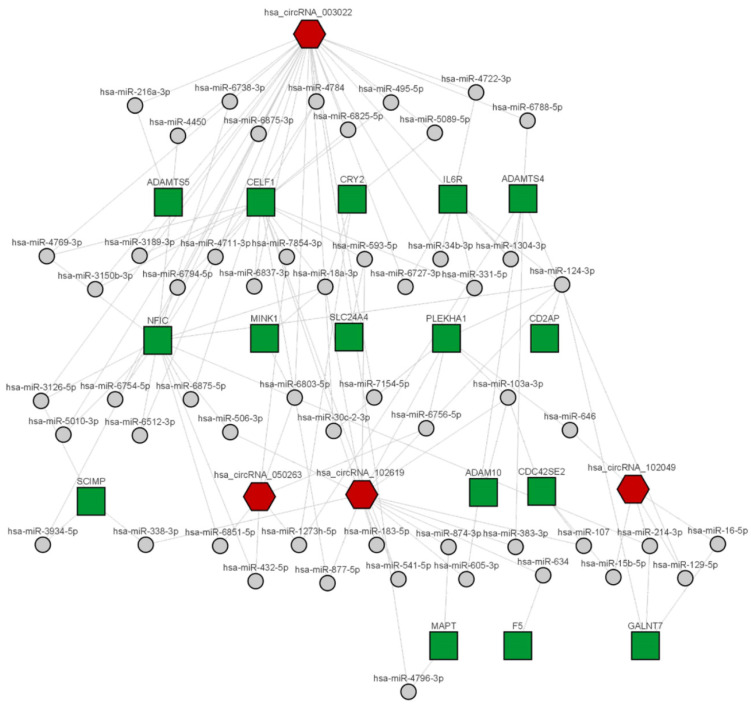
Network of circRNA-miRNA-mRNA for selected targets. CircRNAs are represented as red hexagons, miRNAs as grey dots, and mRNAs as green squares.

To infer a possible biological function of each circRNA, a list of all experimentally validated protein targets of the corresponding microRNAs was initially collected. The number of target proteins in the mirTarBase database [8] for each circRNA (after removing redundancies) ranged from a few dozen (90 for has_circRNA_100837) to a few thousand (3433 for hsa_circRNA_003022). Results were summarized in a Venn diagram in Figure S2. A series of biological annotations were then investigated for each list of the target proteins corresponding to each circRNA. Results from the enrichment statistical analysis focused on the tissue expression of the proteins (as annotated in the Uniprot database), on the associated Biological Processes (as described in the Gene Ontology) or pathways (from the KEGG and Reactome databases) are reported in Figure 6 and Figure 7. We observed that the target proteins (via the intermediate role of microRNAs) of all four investigated circRNAs were prevalent and statistically enriched in the brain. In particular, the targets of hsa_circRNA_003022 resulted significantly over-represented in the amygdala (188 proteins, *p*-adjusted < 0.05), while the targets of hsa_circRNA_102619 were overrepresented in the hippocampus (83 proteins, *p*-adjusted < 0.05), which are both brain tissues affected by AD (Figure 7). Moreover, the target proteins of the four differentially expressed circRNAs are involved in a variety of pathological processes related to Alzheimer’s disease. These included the development of the nervous system (GO: 0007399), the cellular response to nerve growth factor stimuli (GO: 1990090), protein phosphorylation (GO: 0006468), the negative regulation of apoptosis (GO: 0043066), TGF-beta signaling (GO: GO:0007179), the cytokine-mediated signaling pathway (GO:0019221) (Figure 8).

Besides, pathways (KEGG and Reactome) enrichment analysis revealed that dysregulated circRNAs were involved (via the intermediate roles of microRNAs and corresponding target proteins) in AD-related pathways (e.g., neurotrophin signaling pathway, dopaminergic synapse, apoptosis, mTOR, AMPK, and MAPK signaling pathway). The 50 most interesting pathways are shown in Figure 8. The complete lists of all 189 functional terms resulting from the pathways’ enrichment analysis, together with corresponding *p*-values, are available in Table S2.

Moreover, we carried out a further analysis targeting the main genes involved in Alzheimer’s Disease [13]. We found that has_circRNA_102619 target proteins (via the circRNA-miRNA-mRNA regulatory network) were significantly enriched (Fisher’s exact test, genes = 16, *p*-value = 0.06) in the collection of AD-associated genes. In particular, the 16 genes involved were: “ADAM10”, “ADAMTS4”, “CD2AP”, “CDC42SE2”, “CELF1”, “CRY2”, “F5”, “GALNT7”, “IL6R”, “MAPT”, “MINK1”, “NFIC”, “PLEKHA1”, “SCIMP”, “SLC24A4”, “TP53INP1” (Figure 8).

## 3. Discussion

The interest in the role of circRNAs in the physiology and pathology of CNS has been steadily increasing in the last few years [10,14]. Some studies reported that circRNAs could play an important role in AD [8,15], even as possible biomarkers [8]. However, to our knowledge, only one paper was published investigating a profile of plasmatic circRNAs in AD [16], while no studies are available on subjects with MCI. The study reported that 15 circRNAs were significantly higher in participants with AD compared to HCs from two different populations (GSE161199 and PRJNA574438). This lack of data prompted us to assess the profile of plasma circRNAs expression in people with AD and HCs, and explore it in a small population of MCI subjects. Our microarray analysis of 13,617 different probes identified some differentially expressed circRNAs, 10 of which showed a good level (*p* < 0.05) of significance and a high fold change and raw intensity. Clustering analysis showed a different circRNAs expression profile between people with AD and controls. In this analysis, only one HC had results differing from the remaining HC group and similar to people with AD. 

We reanalyzed the six dysregulated circRNAs out of the profiled circRNAs, using qRT-PCR, in a larger population of AD and HC, including also five subjects with MCI. The analysis confirmed the upregulation of hsa_circRNA_050263, hsa_circRNA_403959, and hsa_circRNA_003022 both in participants with AD and MCI compared to HCs, and the downregulation of hsa_circRNA_102049 and hsa_circRNA_102619 only in participants with AD. Interestingly, hsa_circRNA_403959 was significantly overexpressed in MCI than in both AD and HC subjects. Further studies on these differentially expressed circRNAs could lead to identifying novel biomarkers for the early diagnosis of Alzheimer’s disease and the prediction of conversion from MCI to dementia.

These five circRNAs are localized within the ATP13A1, BRAF, PITRM1, TADA2A, and NOL10 gene loci, respectively. ATP13A1, encoding for a lysosomal ATPase was correlated with Parkinson’s Disease [17,18]. BRAF mutations represent a diagnostic criterion for melanoma [19], but recently it was associated with AD [20]. PITRM1 has a role in mitochondrial dysfunction and neurodegeneration. Mitochondria of the temporal lobe showed a PITRM1 reduced activity in patients with AD [21]. Although TADA2A- derived circRNAs have different roles in tumors [22,23], the TADA2A protein seems to interact with the A53T variant of α-synuclein that was preferentially retained in the cellular nucleus. The complex modulates acetylation levels on H3 and H4 histones [24]. Then, these genes are associated with neurological disorders in literature, except for NOL10

We also investigated all microRNAs potentially interacting with the studied circRNAs. In particular, some of them resulted in being targeted by more than one circRNA. Specifically, hsa-miR-18a-5p, hsa-miR-18b-5p, hsa-miR-149-5p and hsa-miR-30e-5p could be regulated by either circRNA_102619 and circRNA_003022; hsa-miR-197-5p by circRNA_102619 and circRNA_102049; hsa-miR-6871-5p, hsa-miR-6751-5p, hsa-miR-6803-5p and hsa-miR-194-3p could be regulated by circR-NA_102049 and circRNA_003022; circRNA_050263 and circRNA_003022 could target hsa-miR-622, hsa-miR-21-3p; hsa-miR-592, hsa-miR-516a-3p, hsa-miR-516b-3p and hsa-miR-4441. Neither circRNA_100837 nor circRNA_403959 show any common interactors (Figure 5). 

Therefore, the four circRNAs associated with the most target miRNA-target were chosen to perform further analysis to investigate the biological function associated with the corresponding gene targets. The GO enrichment analysis showed that the investigated circRNAs are involved in the development of the nervous system, and in the cellular response to nerve growth factor stimuli, protein phosphorylation, apoptotic processes, and inflammation pathways, all of which are processes related to the pathology of AD. Moreover, the KEGG pathway analysis reported that the differentially expressed circRNAs were strongly associated with the regulation of gene pathways that participate in the neurotrophin signaling pathway, and the dopaminergic synapse, apoptosis, mTOR, AMPK, and MAPK signaling pathways, all involved in AD. 

An interesting analysis of a restricted number of genes known to be related to AD [13] showed that hsa_circRNA_102619 enriched with its target proteins in this specific AD-associated collection of genes. Among these potential targets, we found ADAM10 and MAPT (Figure 8). The proteolytic processing of APP by ADAM10 produces the sAPPα secreted fragment that has neuroprotective and neurotrophic properties so that an increase in ADAM10 activity could be potentially therapeutic for AD. MAPT encodes for Tau, one of the main proteins involved in AD. Interestingly, hsa_circRNA_102619 was the main circRNA associated with the hippocampus, as shown by tissue expression analysis (Figure 8).

Several of the circRNA-associated miRNAs are expressed in the brain, with relatively defined roles in brain development (miR-18a, miR-18b [25,26], miR-30 [27,28], miR-592 [29,30]) or adult brain (miR-18a [31,32], miR-149 [33,34,35], miR-30 [36,37], miR-21 [28,38], miR-592 [39]. Some of these were investigated in the field of dementias and neurodegenerative diseases. MiR-18a seems to regulate the BARHL1-ER1 axis, involved in neural death and apoptosis, cerebral development, behavior, and sensorial perceptions [40]. Additionally, miR-18a may have a role in abnormal gene expression detected in the senescence-accelerated mouse-prone 8 (SAMP8) model of sporadic AD [41]. MiR-149-5p may regulate Aβ1-42 deposition and have neuroprotective effects by directly targeting BACE1 [42]. MiR-149-5p also has a role in Parkinson’s disease, particularly in the prevention of cellular death and rupture of the blood-brain barrier [43]. MiR-30a was upregulated in PSEN1 mutation carriers affected by AD respect to healthy PSEN1 mutation carriers, in the same Chinese family [44].

Among studies investigating the roles and the importance of non-coding RNA in neurodegenerative diseases, Dube and colleagues [45] reported data on cortical circRNAs expression observing an association between circRNA levels and AD, and its clinical and neuropathological severity. 

One of the first circRNAs to be characterized was CDR1as associated with ubiquitin-protein ligase A (UBE2A) having a relevant role in clearing amyloid peptides, and it was found depleted in the brain of subjects with AD [10]. Moreover, circAβ-a was also identified, which is a circRNA derived from the coding region of the APP gene. It may be translated, within the brain, into the newly discovered Aβ175 polypeptide, suggesting a new Aβ pathway [15,46]. However, research on circRNAs in AD is still scarce [8]. 

CircRNAs are highly stable molecules for their resistance to exonucleases. They are found in extracellular vesicles and plasma samples [6,45]. For these reasons, they have potential for diagnostic purposes. A recent study observed that changes in circRNAs expression precede substantial symptom onset. This finding, combined with the stability of these molecules in plasma and enrichment in extracellular vesicles, supports the utility of circRNAs as peripheral biomarkers of pre-symptomatic and symptomatic AD.

Our data also showed some interesting differences in the expression of some circRNAs between males and females. Some sex-specific biomarkers of dementia have been investigated in the last years [47,48], and recent studies have suggested that microRNA could be included among them [49], probably along with circRNAs. Shortly, considering the gender of the study participants will allow for the identification of further information on diagnosis, disease progression, and response to possible treatment approaches tailored to individual patients. In this context, sex-specific circRNAs could be useful in identifying patient subgroups. This study has an important limitation. We investigated a small sample of the population. In particular, results from the preliminary study on MCI were obtained from only five participants. Further studies should be carried out to characterize the expression profile and function of circulating circRNAs, in particular exosomal circRNAs, to explore the development of novel biomarkers of preclinical and clinical AD.

## 4. Materials and Methods

### 4.1. Recruited Population

A total of 26 patients with AD, 29 HC, and five subjects with MCI (see Table 1) were consecutively recruited from CCDDs of Sapienza University in Rome and IRCCS Neuromed in Pozzilli, from 10 September 2019 to 12 December 2020. The ethics committees of both institutes approved the study, and informed consent was signed by all participants. 

Alzheimer’s disease was defined according to the DSM-IV and NINCDS-ADRDA criteria [50]. Controls were enrolled among healthy volunteers, including spouses and non-blood-related relatives, who underwent a neurological assessment of both their cognitive and functional status. Inclusion criteria: male and female participants aged 50–85 years. Exclusion criteria: mixed dementia, secondary dementias with a history of stroke or other severe cardiovascular diseases, neuroimaging evidence of other potential causes of cognitive decline, Parkinson’s disease, or Parkinsonism. All patients underwent laboratory tests to rule out other dementias, and brain imaging (magnetic resonance or computed tomography). Participants with MCI were diagnosed using the criteria described by Petersen [51]. Healthy controls were enrolled among family members or caregivers unrelated to the patients.

All participants underwent clinical and neurological assessment, including the administration of the Mini-Mental State Examination (MMSE).

### 4.2. Sample Preparation

The plasma was obtained from whole blood collected in EDTA tubes, by centrifugation at 2500 rpm for 15 min at 4 °C, taking care not to shake the buffy coat. After aliquoting, the tubes were stored in a −80 °C freezer. Total plasma RNA, including small RNAs, was isolated from miRNeasy Mini kit (QIAGEN, Hilden, German). The concentrations of the RNA samples were measured by NanoDrop ND-1000 (Thermo Fisher Scientific, Waltham, MA, USA).

### 4.3. SIMOA analysis

Human Simoa Neurology 3-Plex A (N3PA) Advantage kit was carried out to evaluate levels of total-tau, amyloid-β40, and amyloid-β42, while Simoa *p*-tau181 Advantage kit was used to measure p-tau181 (Quanterix, Billerica, MA, USA). Each sample was measured in duplicate and two control samples added to each plate were used for each analyte. All measurements were performed at Quanterix on a single molecule assay (Simoa) biomarkers detection system.

### 4.4. Microarray Analysis

Sample labeling and array hybridization were performed according to the manufacturer’s protocol (Arraystar Inc., Rockville, MD, USA). Total RNAs were digested with Rnase R (Epicentre, Inc., Madison, WI, USA) to remove linear RNAs and enrich circRNAs. Then, the samples were amplified and transcribed into fluorescent cRNA by random priming (Arraystar Inc.). The labeled cRNAs were purified by RNeasy Mini Kit (QIAGEN) and their concentration and specific activity (pmol Cy3/μg cRNA) was measured by NanoDrop ND-1000 (Thermo Fisher Scientific) Then, 1 μg of each labeled cRNA was fragmented by heating 60 °C for 30 min after the addition of 5 μL of 10 × Blocking Agent and 1 μL of 25 × Fragmentation Buffer. To complete sample preparation for microarrays, 25 μL of 2 × Hybridization buffer was added to the labeled cRNA. Thus, 50 μL of sample mixture was dispensed into the gasket slide and assembled to the circRNA expression Arraystar Human circRNA Array V2 (8×15K, Arraystar Inc.) microarray slide. The slides were incubated for 17 h at 65 °C in an Agilent Hybridization Oven. The hybridized arrays were washed, fixed, and scanned using the Agilent Scanner G2505C (Agilent Technologies, USA). The normalization of circRNA microarray data followed the quantile method. 

### 4.5. Quantitative Real-Time PCR Analysis

RNA was retro-transcribed using First Strand cDNA Synthesis (Thermo Fisher Scientific) according to the manufacturer’s protocol. After dilution of cDNA, samples were quantified in triplicates by using specific primers (Arraystar Inc.) described in Table 4 for amplify hsa_circRNA_100837, hsa_circRNA_403959, hsa_circRNA_102619, hsa_circRNA_003022, hsa_circRNA_050263, hsa_circRNA_102049. β-actin was used as endogenous control, as previously described [11,12]. qRT-PCR reactions were performed using Arraystar PCR master mix in an ABI PRISM7500 Real-Time PCR Detection System (Thermo Fisher Scientific). The PCR reaction was obtained with 2 µL of the diluted cDNA template, 5 µL of SYBR^®^ Green master mix, and 1 µL of PCR primer mix. The Real-Time PCR cycles were 95 °C for 10 min, followed by 40 amplification cycles of 95 °C 10 s and 60 °C 1 min. Raw Ct values were normalized through the ∆Ct method using as a reference the endogenous control. 

### 4.6. CircRNA-microRNA-mRNA Network

Bioinformatics analyses were performed to identify potential microRNA targets on each differentially expressed circRNA. Two different prediction tools were applied: TargetScan [52] and miRanda [53] software. The list of putative circRNA-associated microRNAs was compiled for each circRNA under examination (Table S1). For each microRNA, all experimentally validated target genes in the miRTarBase database (release 8.0) [54] were collected using the miRWalk platform [55] and considered for further analysis. 

After, a Functional Enrichment Analysis was performed. The biological functions of each circRNA were inferred by analyzing the protein-coding genes resulting as potential final targets in the regulatory network of circRNA-miRNA-mRNA. Over-represented biological terms (which have more genes than expected by chance) were identified by using the DAVID web server [56] with the entire human proteome as a reference and querying the following categories: Gene Ontology (GO) Biological Processes (BP) [57]; pathways collected in KEGG [58] and Reactome [59] databases; and UniProt [60] Keyword protein annotations related to Tissue Expression. Only biological categories with Benjamini-Hochberg adjusted *p*-value ≤ 5 × 10^−2^ were considered statistically enriched [61]. Results for each investigated group are shown as heat maps, produced using the ggplot2 R-package: https://ggplot2.tidyverse.org (accessed on 22 February 2022) with the color scale representing the corrected *p*-values. Cytoscape v3.9.0 [62] has been utilized to visualize circRNA-miRNA-mRNA networks. 

### 4.7. Statistical Analysis

Continuous data were summarized by mean and standard error or standard deviation. Categorical variables were described by frequency and percentage distributions. Comparisons between experimental groups were assessed by the one-way ANCOVA test adjusted for sex and age. *t*-Test or Mann–Whitney test for continuous variables, Fisher’s exact probability test, and Pearson’s chi-square test for categorical variables were also used. Correlation analyses were performed by Pearson’s index test. The level of significance was 0.05 and Bonferroni’s correction was adopted to control for type I error in multiple comparisons. All statistical analyses were carried out by the software SPSS (version 28.0). 

## Figures and Tables

**Table 1:** Population characterization. All values are expressed as mean ± standard deviation. HC: healthy controls; AD: Alzheimer’s disease; MCI: Mild Cognitive Impairment. MMSE: Mini-Mental State Examination; NA: not available; *p*: *p*-value; n.s.: non-significant.

	Pilot Study			Validation Study			
	HC	AD		HC	AD	MCI	
N.	5	5		24	21	5	
Sex (M/F)	2/3	2/3	*p* = n.s.	10/14	9/12	3/2	*p* = n.s.
Age at the evaluation	73.8 ± 4.3	74.4 ± 2.3	*p* = n.s.	69.9 ± 6.6	74.4 ± 6.1	70.0 ± 7.3	*p* = n.s.
Age at onset	-	66.40 ± 6.5		-	70.6 ± 6.8	66.4 ± 6.5	
MMSE	-	21.4 ± 1.4		-	21.1 ± 1.7	25.6 ± 0.9	
Aβ42/Aβ40		NA			0.04 ± 0.007	0.05 ± 0.001	
Total-tau		NA			2.31± 1.9	2.98 ± 0.6	
p-tau		NA			2.78 ± 0.5	2.37 ± 0.6	

**Table 2:** Up and down regulated circRNAs in AD vs HC. Fold Change cut off: 2.0; *p*-value cut off: 0.05.

CircRNA	*p*-Value	FC (abs)	Regulation	Chrom.	Strand	circRNA_Type	Gene Symbol
hsa_circRNA_100837	0.0037	2.40	up	chr11	−	exonic	STX5
hsa_circRNA_100760	0.0042	2.23	up	chr11	−	exonic	DENND5A
hsa_circRNA_403959	0.0051	2.23	up	chr7	−	exonic	BRAF
hsa_circRNA_001131	0.0122	2.55	up	chr2	−	intronic	TLR5
hsa_circRNA_405788	0.0104	2.38	up	chr19	−	Exonic	CADM4
hsa_circRNA_050263	0.0155	2.19	up	chr19	−	exonic	ATP13A1
hsa_circRNA_003022	0.0180	2.14	up	chr10	−	exonic	PITRM1
hsa_circRNA_407191	0.0126	2.00	up	chr9	+	Sense overlapping	AL161626.1
hsa_circRNA_102750	0.0425	1.55	up	chr2	+	exonic	MEIS1
hsa_circRNA_105042	0.0128	1.90	up	chrX	−	exonic	GAB3
hsa_circRNA_090183	0.0114	1.74	up	chrX	+	exonic	PRRG1
hsa_circRNA_401844	0.0111	1.92	up	chr17	−	exonic	TUBD1
hsa_circRNA_080099	0.0073	1.66	up	chr7	−	exonic	MYO1G
hsa_circRNA_004907	0.0316	1.60	up	chr10	+	exonic	ZEB1
hsa_circRNA_101222	0.0343	1.57	up	chr13	−	exonic	TPTE2
hsa_circRNA_002165	0.0340	1.60	up	chr6	−	exonic	SRPK1
hsa_circRNA_003022	0.0380	2.14	up	chr10	−	exonic	PITRM1
hsa_circRNA_003574	0.0104	1.88	up	chr20	+	exonic	GID8
hsa_circRNA_102885	0.0273	1.77	up	chr2	−	exonic	SATB2
hsa_circRNA_104671	0.0283	1.82	up	chr8	−	exonic	UBR5
hsa_circRNA_103618	0.0255	1.78	up	chr4	−	exonic	ARAP2
hsa_circRNA_104220	0.0109	1.67	up	chr6	+	exonic	PCMT1
hsa_circRNA_101752	0.0181	1.56	up	chr16	−	exonic	LOC100271836
hsa_circRNA_100759	0.0206	1.72	up	chr11	−	exonic	DENND5A
hsa_circRNA_102049	0.0279	2.11	down	chr17	+	exonic	TADA2A
hsa_circRNA_102619	0.0036	2.00	down	chr2	−	exonic	NOL10
hsa_circRNA_102645	0.0454	1.62	down	chr2	−	exonic	HADHA

**Table 3:** Mean ∆Ct values and the respective standard errors of each circRNA, for HCs, AD, and MCI participants. In the last columns, *p*-values and *p*-values adjusted for sex and age-related to each comparison are reported.

circRNA	Mean Value (∆Ct)		Standard Error		*p*_Values	*p* Values Adjusted
Hsa_circRNA_102049	HC	0.000737	0.000048	HC/AD	<0.001	<0.001
	AD	0.000404	0.000056	HC/MCI	0.355	0.474
	MCI	0.000549	0.000077	AD/MCI	0.687	0.687
Hsa_circRNA_050263	HC	0.000750	0.000093	HC/AD	<0.001	<0.001
	AD	0.002293	0.000120	HC/MCI	<0.001	<0.001
	MCI	0.001945	0.000179	AD/MCI	0.494	0.668
Hsa_circRNA_102619	HC	0.001418	0.000215	HC/AD	0.003	0.006
	AD	0.000585	0.000082	HC/MCI	0.430	0.548
	MCI	0.000839	0.000228	AD/MCI	0.217	1.000
Hsa_circRNA_403959	HC	0.001136	0.000114	HC/AD	0.013	0.026
	AD	0.001559	0.000116	HC/MCI	<0.001	<0.001
	MCI	0.006260	0.003491	AD/MCI	0.001	0.001
Hsa_circRNA_003022	HC	0.000295	0.000085	HC/AD	0.015	0.094
	AD	0.000695	0.000106	HC/MCI	0.015	0.016
	MCI	0.000954	0.000227	AD/MCI	0.777	0.451
Hsa_circRNA_100837	HC	0.000364	0.000039	HC/AD	0.389	0.072
	AD	0.001407	0.000555	HC/MCI	0.070	0.055
	MCI	0.002972	0.002349	AD/MCI	0.513	1.000

**Table 4:** PCR primers set (Arraystar Inc.) used for qRT-PCR validation.

Gene Name	Sequence (5′ to 3′)	Tm (°C)	Length of Product (bp)
hsa_circRNA_100837	Forward:5′ AAGCAGTGGAAATTGAAGAGC3′ Reverse:5′ GCTGGCTTATTTGTCTGGATT3′	60	67
hsa_circRNA_403959	Forward:5′ AGAAGACAGGAATCGAATGGACT3′ Reverse:5′ CAGGTAATGAGGCAGGGGG3′	60	96
hsa_circRNA_102619	Forward:5′ GGGCATCTATTACATTCCATTCT3′ Reverse:5′ ATTATTCTCCGCAGCATCAGT3′	60	95
hsa_circRNA_003022	Forward:5′ GATGAAGGGAGCGTTTACAGA3′ Reverse:5′ GGGAACAGATGTCACCTAGCA3′	60	192
hsa_circRNA_050263	Forward:5′ CAAGCTCTCATCCATCCAGTG3′ Reverse:5′ ATGGGCGTACTCTCGTCCTC3′	60	73
hsa_circRNA_102049	Forward:5′ CACAGCCATTCCATTTCACTACT3′ Reverse:5′ CAAAGCCACAGTCCATCACAG3′	60	105
β-actin	Forward:5′ GTGGCCGAGGACTTTGATTG3′ Reverse:5′ CCTGTAACAACGCATCTCATATT3′	60	73

## Data Availability

The dataset generated during the current study will be available from the corresponding author (P.P.) upon reasonable request.

## Supplementary Materials

The following supporting information can be downloaded at: https://www.mdpi.com/article/10.3390/ijms232113232/s1.
