# Supplementary material for: A Plasma Circular RNA Profile Differentiates Subjects with Alzheimer’s Disease and Mild Cognitive Impairment from Healthy Controls
Source: Int J Mol Sci. 2022 Oct 31;23(21):13232. doi: 10.3390/ijms232113232 (PMC9658433; doi:10.3390/ijms232113232)

Supplementary Figure S1. (A) The X and Y values on Scatter Plot referred the average normalized signal values of the samples (log2 scaled). The green lines represent the fold change. The CircRNAs above the top green line and below the bottom green line indicated more than 1.5-fold change of circRNAs between the two compared samples. (B) The vertical lines of the Volcano Plot correspond to 1.5-fold up and down, while the horizontal one represents a p-value of 0.05. The red point in the plot represents the differentially expressed circRNAs with statistical significance. (C) Boxes in the Boxplot included the median (middle horizontal line) and the interquartile (up and down horizontal lines) of the normalized intensity values. (D) Hierarchical clustering of differentially expressed circRNAs. Green: low intensity, black: medium intensity, red: strong intensity

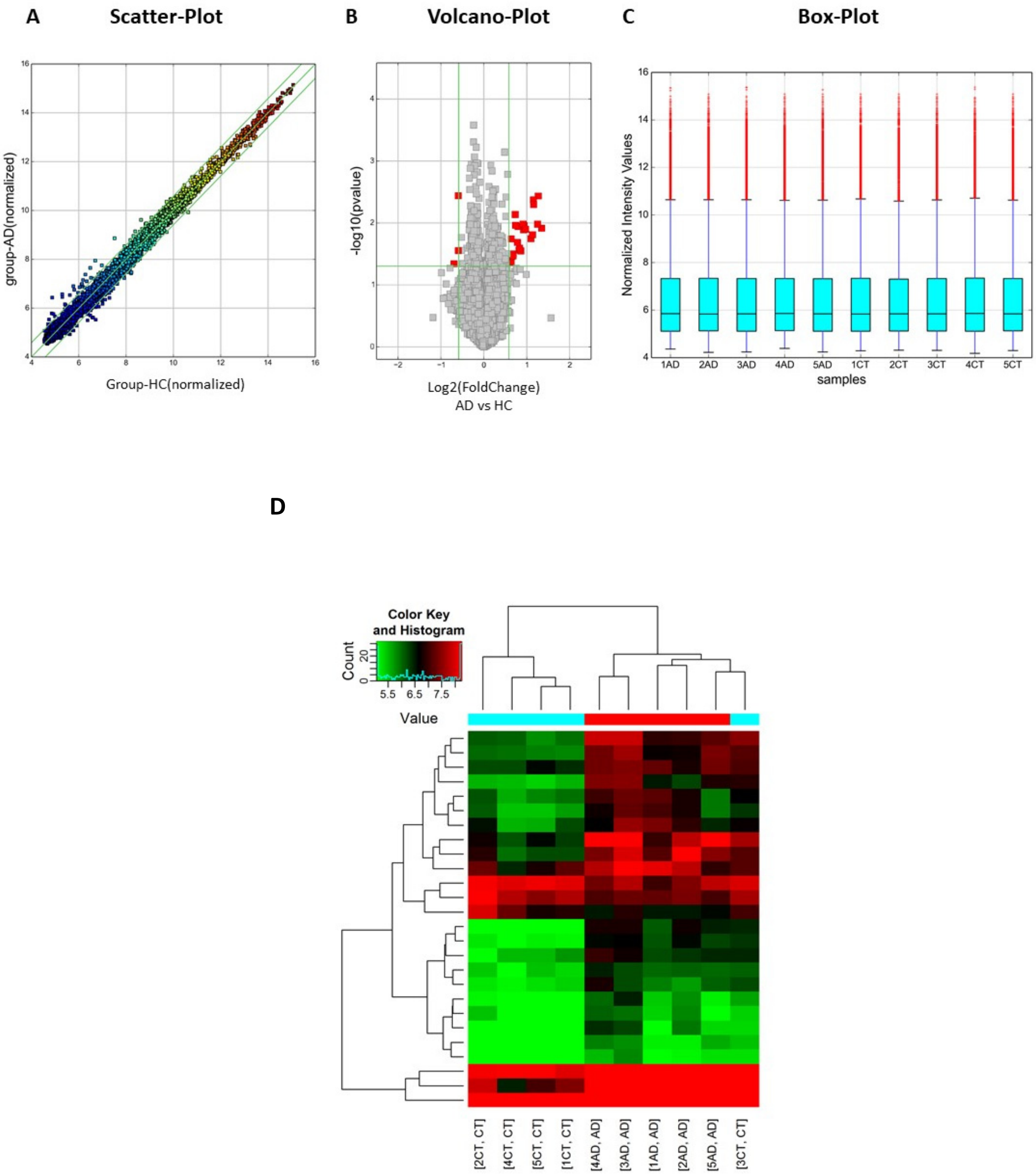

Supplement: Supplementary file 1 [file ijms-23-13232-s001.zip › Figure S1.pdf]
