# Supplementary material for: A Plasma Circular RNA Profile Differentiates Subjects with Alzheimer’s Disease and Mild Cognitive Impairment from Healthy Controls
Source: Int J Mol Sci. 2022 Oct 31;23(21):13232. doi: 10.3390/ijms232113232 (PMC9658433; doi:10.3390/ijms232113232)
Supplement: Supplementary file 1 [file ijms-23-13232-s001.zip › Supplementary Materials caption.pdf]

(A) Scatter plot. Each colored dot represents a different circRNA-corresponding signal obtained from the microarray. The averages of normalized signal values, scaled log2, from HC and AD groups are graphed respectively on the X- and Y-axes. The fold change (FC) values are represented with green lines. Specifically, the top green line corresponds to FC = 1.5, while the bottom one to FC= -1.5. Thus, differentially expressed circRNAs that have a fold change greater than ± 1.5 are represented with dots above and below these lines. (B) Volcano plot. It graphs log2 scaled Fold Change between AD and HC groups (X-axis) and -lo10 scaled *p* values (Y-axis). Vertical green lines represent FC ± 1.5, horizontal one refers to *p*-value = 0.05, and squares correspond to circRNA' signals. Red squares represent significant differential expressed circRNAs (FC > ±1.5; $p < 0.05$). (C) Box plot. For the normalized intensity values, the median is graphed with the middle horizontal line in every box, while the lines at the top and bottom of the box represent the interquartile values. (D) Hierarchical cluster. The intensity of the differential expressed circRNAs is indicated by colors: green for low, black for intermediate, and red for strong intensity. Figure S2. Venn diagrams showing the number of target proteins (via the intermediate role of microRNAs) of each circRNA, their overlaps, and specificities; target proteins are those contained in miRTarBase database (release 8.0); Table S1: List of predicted miRNAs as targets of each circRNA; Table S2: KEGG and REACTOME pathway enrichment analysis of proteins target of each circRNA.
