# Supplementary figures and images for: A Plasma Circular RNA Profile Differentiates Subjects with Alzheimer’s Disease and Mild Cognitive Impairment from Healthy Controls
Source: Int J Mol Sci. 2022 Oct 31;23(21):13232. doi: 10.3390/ijms232113232 (PMC9658433; doi:10.3390/ijms232113232)

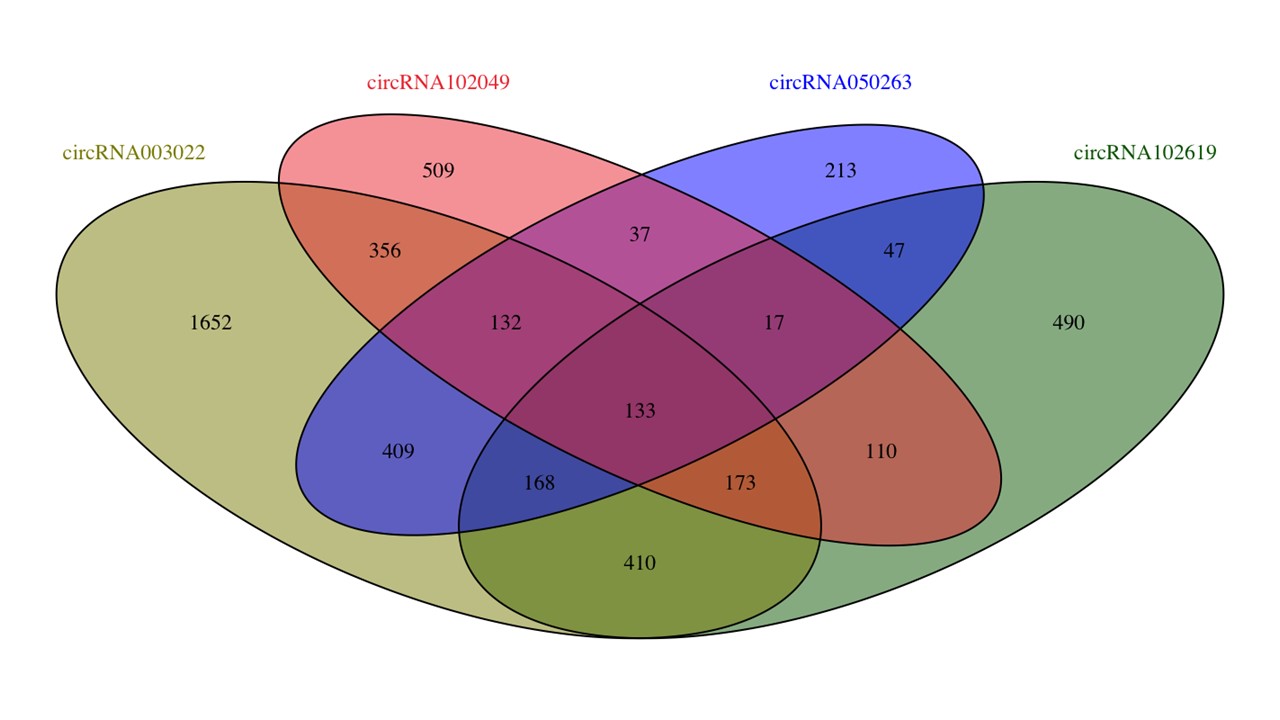

Supplement: Supplementary file 1 [file ijms-23-13232-s001.zip › Figure S2.jpg]
